# Is the leptin/BMI ratio a reliable biomarker for endometriosis?

**DOI:** 10.3389/fendo.2024.1359182

**Published:** 2024-03-19

**Authors:** Aleksandra Zyguła, Anna Sankiewicz, Agata Sakowicz, Ewa Dobrzyńska, Agnieszka Dakowicz, Grzegorz Mańka, Mariusz Kiecka, Robert Spaczynski, Piotr Piekarski, Beata Banaszewska, Artur Jakimiuk, Tadeusz Issat, Wojciech Rokita, Jakub Młodawski, Maria Szubert, Piotr Sieroszewski, Grzegorz Raba, Kamil Szczupak, Tomasz Kluza, Marek Kluza, Piotr Pierzyński, Cezary Wojtyla, Michal Lipa, Damian Warzecha, Miroslaw Wielgos, Krzysztof Cendrowski, Ewa Gorodkiewicz, Piotr Laudanski

**Affiliations:** ^1^ OVIklinika Infertility Center, Warsaw, Poland; ^2^ Bioanalysis Laboratory, Faculty of Chemistry, University of Bialystok, Bialystok, Poland; ^3^ Department of Medical Biotechnology, Medical University of Lodz, Lodz, Poland; ^4^ Chair and Department of Obstetrics, Gynecology and Gynecological Oncology, Medical University of Warsaw, Warsaw, Poland; ^5^ Department of Rehabilitation, Medical University of Bialystok, Bialystok, Poland; ^6^ Angelius Provita Hospital, Katowice, Poland; ^7^ Center for Gynecology, Obstetrics and Infertility Treatment Pastelova, Poznan, Poland; ^8^ Collegium Medicum, University of Zielona Gora, Zielona Gora, Poland; ^9^ Gynecological Obstetric Clinical Hospital of Poznan University of Medical Sciences, Minimally Invasive Gynecological Surgery, Poznan, Poland; ^10^ Chair and Department of Laboratory Diagnostics, Poznan University of Medical Sciences, Poznan, Poland; ^11^ Department of Reproductive Health, Institute of Mother and Child in Warsaw, Warsaw, Poland; ^12^ Department of Obstetrics and Gynecology, Central Clinical Hospital of the Ministry of Interior, Warsaw, Poland; ^13^ Department of Obstetrics and Gynecology, Institute of Mother and Child in Warsaw, Warsaw, Poland; ^14^ Collegium Medicum, Jan Kochanowski University in Kielce, Kielce, Poland; ^15^ Clinic of Obstetrics and Gynecology, Provincial Combined Hospital in Kielce, Kielce, Poland; ^16^ Department of Gynecology and Obstetrics, Medical University of Lodz, Lodz, Poland; ^17^ Department of Surgical Gynecology and Oncology, Medical University of Lodz, Lodz, Poland; ^18^ Department of Fetal Medicine and Gynecology, Medical University of Lodz, Lodz, Poland; ^19^ Clinic of Obstetric and Gynecology in Przemysl, Przemysl, Poland; ^20^ Medical College of Rzeszow University, Rzeszow, Poland; ^21^ Department of Gynecology, Gynecology Oncology and Obstetrics, Institute of Medical Sciences, Medical College of Rzeszow University, Rzeszow, Poland; ^22^ Women’s Health Research Institute, Calisia University, Kalisz, Poland; ^23^ Departament of Obstetrics and Perinatology National Medical Institute of the Ministry of Interior and Administration, Warsaw, Poland; ^24^ City South Hospital, Warsaw, Warsaw, Poland; ^25^ Department of Biomedical Fundamentals of Development and Sexology, Faculty of Education, University of Warsaw, Warsaw, Poland; ^26^ Premium Medical Clinic, Warsaw, Poland; ^27^ Medical Faculty, Lazarski University, Warsaw, Poland

**Keywords:** endometriosis, leptin, plasma, peritoneal fluid, infertility

## Abstract

**Background:**

The aim of this study was to analyze the concentration of leptin in peritoneal fluid and plasma and to assess their role as potential biomarkers in the diagnosis of endometriosis.

**Materials & methods:**

Leptin adjusted for BMI (leptin/BMI ratio) was measured using surface plasmon resonance imaging (SPRI) biosensors. Patients with suspected endometriosis were included in the study. Plasma was collected from 70 cases, and peritoneal fluid from 67 cases. Based on the presence of endometriosis lesions detected during laparoscopy, patients were divided into a study group and a control group (patients without endometriosis).

**Results:**

Leptin/BMI ratio in plasma did not differ between women with endometriosis and the control group (0.7159 ± 0.259 vs 0.6992 ± 0.273, p= 0,7988). No significant differences were observed in peritoneal leptin/BMI ratio levels in patients with and without endometriosis (0.6206 ± 0.258 vs 0.6215 ± 0.264, p= 0,9896). Plasma and peritoneal leptin/BMI ratios were significantly lower in women with endometriosis - related primary infertility compared to women with endometriosis without primary infertility (0.640 ± 0.502 vs 0.878 ± 0.623, p < 0.05). The difference was observed in case of primary infertility, but not in terms of the secondary one. No significant differences were noted between leptin/BMI ratio in the proliferative phase and the secretory phase (0.716 ± 0.252 vs 0.697 ± 0.288, p= 0,7785).

**Conclusion:**

The results of present study do not support the relevance of leptin concentration determination as a biomarker of the endometriosis. Due to the limited number of samples in the tested group, further studies are needed to confirm its role.

## Introduction

Endometriosis is a common chronic gynecological disease characterized by the presence of endometrium – like tissue outside the uterine cavity (1). Symptomatic endometriosis is estimated to affect 5 - 15% of women, with around half of women experiencing fertility problems (2–4). The varying extent of the disease (peritoneal endometriosis, ovarian endometrioma, deep infiltrating endometriosis) contributes to a wide array of clinical symptoms such as infertility, chronic pelvic pain, dysmenorrhea, dyspareunia, anxiety, and depression (5). The nonspecific nature of these symptoms makes early diagnosis of endometriosis challenging, often leading to diagnostic delays of 7-12 years (6–8). Consequently, extensive research efforts are underway to identify reliable and non-invasive biomarkers for this disease.

Despite numerous studies have been conducted, the potential pathogenesis of endometriosis remains elusive and requires further elucidation. Sampson’s widely recognized theory suggests that retrograde menstruation is a contributing factor (9). However, while approximately 90% of menstruating women experience retrograde menstruation, only around 10% develop endometriosis from this backward flow of menstrual fluid (10). Other theories emphasize the involvement of genetic, environmental, and immunological factors in predisposing the development of the disease (3). Disturbed immunoregulatory mechanisms, accompanied by various inflammatory markers such as immune cells, cytokines, chemokines, metalloproteinases, cathepsin, and other immunologically-related substances, have been implicated in the development, invasion, and angiogenesis of ectopic lesions (11–18). However, none of these theories fully explain the complexity underlying the pathogenesis of endometriosis.

Relatively little is known about the molecular background of metabolic processes involved in the development of this pathology (19). More recently, molecule such as leptin, which is known to regulate long-term energy balance, has been evaluated as a potential biomarker for endometriosis. Leptin a product of *ob* gene, is a peptide hormone produced by white adipose tissue (WAT). It is also synthesized by other tissues, including the endometrium, placenta and ovary (20–24). Leptin concentration are positively correlated with WAT mass (21). In the plasma, this hormone exists in two forms, free or bound to leptin-binding proteins. Its actions are mediated via binding to leptin receptors (LepRb/ObR, member of class I cytokine receptor family), located in the hypothalamus and various peripheral tissues (21, 22, 25, 26). Leptin was initially described as a key regulator of food intake and modulation of energy expenditure. Subsequently it has been shown, that leptin exert pleiotropic actions by regulating immune homeostasis, promoting neoangiogenesis and reproduction, so it is thought that this molecule may also play a role in pathogenesis of endometriosis (3, 5, 21, 26, 27).

Studies revealed the direct positive correlation between leptin levels and obesity, measured by Body Mass Index (BMI) (28). For this reason, in order to eliminate factors that may influence leptin levels, such as body weight, leptin/BMI ratio was used for measurements.

Interestingly, while leptin, primarily produced by adipocytes, might suggest a higher occurrence of endometriosis in women with an increased BMI, there exists an inverse correlation between BMI and the prevalence of endometriosis (29, 30). Contrary to the assumption, obesity does not act as a protective factor against endometriosis; instead, an elevated BMI may be associated with more severe forms of the disease. This may be due to the role played by leptin, which not only regulates energy balance but also exhibits pro-inflammatory and angiogenic function (31).

The aim of this study was to compare leptin/BMI ratio in biological fluids (plasma and peritoneal fluid) between women diagnosed with endometriosis and a control group, using SPRi biosensors. Furthermore, the study assessed the potential role of leptin as a biomarker of endometriosis.

Surface plasmon resonance imaging (SPRi) is at the forefront of optical sensing. This technique real-time, direct, and ‘label-free’ detection and monitoring of biomolecular events. It involves a plasmon resonance-imaging-supporting metal surface which combines light energy with an electromagnetic field on cells and surface-associated leptin. The main advantage of the SPRi is specificity of molecule determination as well as highly sensitive measurement of the refractive index. This characteristic makes it ideal for the quantification of biomolecules, such as different types of proteins (32–38). Applying the stationary SPRi version in a model investigation and in the determination of various biomarkers in real clinical samples has demonstrated that this technique is suitable for use without signal enhancement or analyte preconcentration (13). Recent reviews highlight SPRi detection as the most promising among surface plasmon-based techniques (32).

## Materials and methods

### Study population

This multicenter, cross-sectional study was conducted across eight Departments of Obstetrics and Gynecology in Poland between 2018 and 2019. Comprehensive recruitment details are provided in our latest publication (39).

All participants, both patients with endometriosis and controls, provide written informed consent, and the study was approved by the Ethics Committee of the Medical University of Warsaw (KB/223/2017).

The study group included individuals aged between 19 and 45 years, who were qualified for planned laparoscopic surgeries due to one or more non-malignant conditions, including infertility, chronic pelvic pain syndrome, ovarian cysts, or suspicion of endometriosis. Exclusion criteria encompassed irregular menstruations cycles (less than 25 days or more than 35), recent hormonal treatment within three months preceding laparoscopy, previous and/or current pelvic inflammatory disease, uterine fibroids, polycystic ovary syndrome, autoimmune comorbidities, malignancies, or any previous history of prior surgical treatment. All patients underwent gynecological examination and vaginal ultrasonography before being referred for surgery. Routine additional radiological examinations to assess extraperitoneal endometriosis were not performed as part of the standard procedure. Each patient was assessed based on the revised American Fertility Society (AFS) classification of endometriosis, complemented by histological examination of collected specimens. Furthermore, all patients completed a World Endometriosis Research Foundation (WERF) clinical questionnaire. As controls, we recruited patients without visible endometriosis during laparoscopy.

The cycle phase was determined based on the last menstrual period and the average length of the menstrual cycle. To ascertain the phases of the menstrual cycle in both women with and without endometriosis, histological dating of eutopic endometrial samples was performed concurrently with the collection of pathological lesions.

The details of the collection and structure of study groups have already been published (39). Briefly, trained gynecologists performed diagnostic laparoscopy on all patients, conducting a comprehensive examination of the uterus, fallopian tubes, ovaries, pouch of Douglas, and pelvic peritoneum. Peritoneal fluid (PF) was aspirated using a Veress needle under direct visualization immediately upon the laparoscope’s insertion to prevent blood contamination. The procedure meticulously followed the standard operating procedures outlined by the Endometriosis Phenome and Biobanking Harmonization Project (40). At all centers, the collected PF underwent centrifugation at 1000g for 10 minutes at 4°C. The resulting supernatant was transferred to fresh 10 ml Sarstedt tubes and divided into 500 ml aliquots. Blood samples were obtained before laparoscopy, always prior to anesthesia, using EDTA 10 ml Sarstedt tubes. Consistent tube types were utilized for both blood and PF across all centers. The time elapsed between sample collection (both PF and plasma) and processing was kept under 45 minutes. Blood samples were centrifuged at 2500g for 10 minutes at 4°C, and then plasma samples were divided into 500 μl aliquots. PF and plasma were stored at -80°C until further use. All samples were transported on dry ice to the Department of Obstetrics and Gynecology in Warsaw, serving as the coordinating center for the study.

Patients diagnosed with endometriosis during laparoscopy were allocated to the corresponding endometriosis stage subgroups (I–IV). Leptin concentration assessment involved the collection of plasma and peritoneal fluid. The procedures adhered to the Endometriosis Phenome and Biobanking Harmonization Project standard operating procedures (40). After exclusion of the outlier results, the final analysis included 70 plasma samples (40 from patients with and 30 from those without) and 67 samples of the peritoneal fluid (38 from patients with and 29 from those without).

### Method of measuring leptin concentration

In present study leptin concentrations in plasma and peritoneal fluid were measured by biosensor SPRI (Surface Plasmon Resonance Imaging) as described in detail by Sankiewicz et al. (41). The base of the biosensor was a gold plate covered with a separating mask of polymer. The first step in preparing the biosensor was to create a cysteamine layer on gold (cysteamine hydrochloride, Sigma-Aldrich, Stieinhem, Germany). The next step was the binding of the leptin capture element, that is rabbit anti-leptin antibody (concertation= 60 ng mL1. pH = 7.4), (Abcam, Cambridge, United Kingdom). A leptin-specific rabbit antibody was immobilized by the formation of a covalent bond between the amine group of cysteamine and the carboxy group of the heavy chain of the antibody. The antibody was activated with the N-ethyl-N’-(3-dimethylaminopropyl) carbodimide (EDC)/N-hydroxysuccimide (NHS) (Sigma-Aldrich, Steinheim, Germany) mixture in carbonate buffer, and then 3 µl of the antibody solution prepared in this way was applied to the active sites (cysteamine layer). The prepared plate was placed in an incubator at 37°C. After 1 hour, the active sites of the biosensor were washed with distilled water and HBS-ES solution. To eliminate the risk of non-specific adsorption, a BSA solution (C = 1 mg/ml) was used. The biosensor prepared in this way, the biosensor was capable of capturing leptin from a solution. The standard solutions of leptin (Abcam, Cambridge, United Kingdom), samples plasma but also peritoneal fluid (5 times diluted with PBS buffer) with a volume of 3 µL were applied on the active sites of the biosensor. After 10 minutes, the surface of the active sites of the biosensor was rinsed with distilled water and HBS-ES solution to remove excess unbound protein.

SPRI measurements were carried out on an apparatus constructed in our laboratory. The SPRi apparatus is composed of a optical part (laser HE-Ne, system of polarizers, lenses, mirror and prism) and detection part (CCD camera connected to computer). A previously prepared biosensor was placed on the prism of the SPRi device and the appropriate angle of incidence of light was selected. The SPR signal was measured at the selected fixed SPR angle. Surface plasmon resonance in the image version examines the change in the intensity of the reflected monochromatic and p-polarized light after applying successive layers of the biosensor. The SPRi signal is proportional to the immobilized mass on the biosensor surface. Signal SPRI are recorded using a CCD camera in the form of images that are subjected to mathematical analysis using a program ImageJ software (version 1.53, National Institutes of Health, NIH). Images are taken twice, for the antibody layer and the analyte layer. Concentrations for the leptin were determined on the basis of calibration curve (in range 0.1–5.0 ng mL1) considering the appropriate dilutions.

### Statistical analyses

Statistical analysis was performed using the statistical package Statistica 13 (TIBCO Software Inc.; 2017). In this study, we assessed variables that adhered to a normal distribution, including age, leptin levels in plasma and peritoneal fluid, and the leptin/BMI ratio in both fluids, confirmed by the Shapiro-Wilk test. Differences between two groups for these variables were analyzed using an independent t-test. Comparisons of leptin levels in plasma and peritoneal fluid within subgroups were conducted using a paired t-test. Results are expressed as mean ± SD.

For variables that did not conform to a normal distribution, specifically BMI, the Mann-Whitney U test was used to assess the significance of differences between groups. In these cases results are expressed as median ± IQR.

When examining differences among three or more groups, we employed an analysis of variance (ANOVA), followed by Duncan’s *post hoc* test for detailed group comparisons. The effect size for these analyses was estimated using the partial eta squared (η²part.) measure.

The contrast analysis was used to highlight patterns of differences between the control and endometriosis groups according to three status of infertility (no infertility, primary infertility and secondary infertility). Contrasts were introduced for each factor separately as follows: Group (1,-1) Infertility (1,-2,1). To explore the correlations between variables, we applied either the simple correlation coefficient (r_xy_) or Spearman’s rank correlation coefficient (r_S_), depending on their distribution of values. For qualitative features, significance of relationships were assessed using Fisher’s exact 2-tailed test, and the effect size for these analyses was estimated with Yule’s coefficient (φ). A significance level α = 0.05 was adopted in this study.

was used for estimate p-value

## Results

The 70 patients i.e. 40 women with diagnosed endometriosis and 30 controls were enrolled to the study. The age of patients ranged from 19 to 45 years, with a mean body mass index (BMI)of 22.3 kg/2 in the study group and 21.58 kg/m^2^ in the control group. No statistically significant difference according age and BMI between groups was found.

According to rAFS classification 27,5% of women were diagnosed as I stage, 27,5% as II stage, 27,5% as III stage, and 17,5% of women as IV stage of endometriosis.

The patient clinical characteristics is presented in **Table 1**.

**Table 1:** Baseline characteristics of participants and leptin levels.

Demographic and anthropometric variables^1^	Endometriosis	Controls	*p-value*
Age [yr] (M ± SD; n)	31.60 ± 5.33; 40	30.87 ± 7.06; 30	0.6459
Body-mass index [kg/m^2^] (Me ± IQR)	22.23 ± 4.20; 40	21.58 ± 5.38; 28	0.7178
rAFS stage of disease %; N			
I	27.50;11	-	-
II	27.50;11	-	-
III	27.50;11	-	-
IV	17.50;7	-	-
Menstrual cycle phase (n; %)			
Proliferative	24; 60%	21; 70%	0.4552
Secretive	16; 40%	9; 30%	
Leptin levels			
Plasma (ng/mL) (M ± SD; n)	15.578 ± 5.162; 40	15.530 ± 6.404; 30	0.9721
Peritoneal fluid (ng/mL) (M ± SD; n)	13.615 ± 5.322; 38	13.576 ± 6.209; 29	0.9785

The mean plasma leptin concentration (ng/mL) were comparable in two groups, 15.578 ± 5.162 ng/mL in women with endometriosis, and 15.530 ± 6.404 ng/mL in the control group.

The levels of leptin in peritoneal fluid (ng/ml) did not differ significantly between the groups, measuring 13.615 ± 5.322 in the study group and 13.576 ± 6.209 in the control group, respectively.

### Leptin/BMI ratio in body fluids

**Figure 1:**
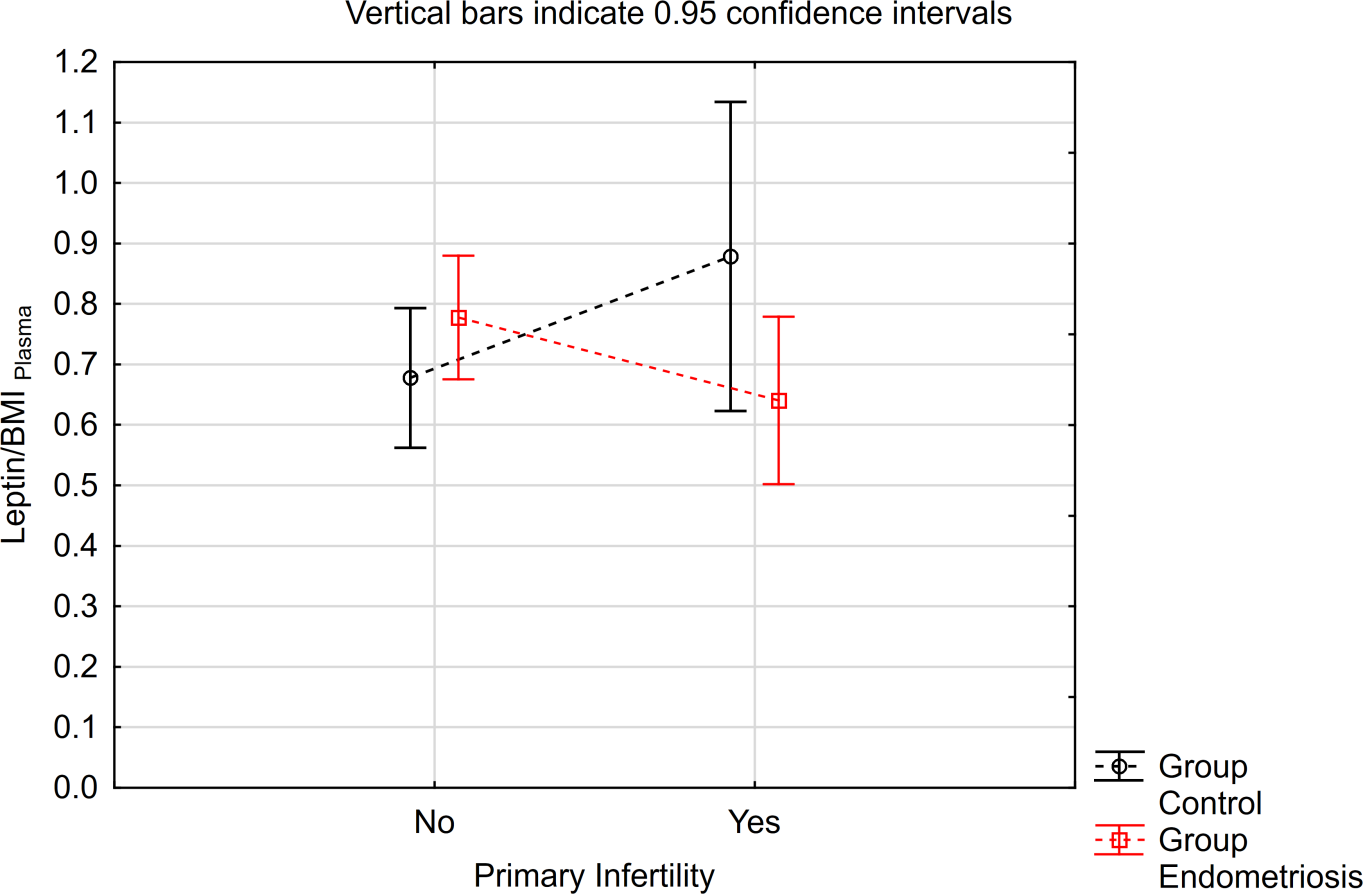
The means of leptin/BMI ratio in plasma depending of endometriosis and primary infertility.

Leptin/BMI ratio in plasma did not show statistical differences between women with endometriosis and the control group, nor between patients with primary infertility (**Table 2**, **Figure 1**). Additionally, the interaction between these factors was not statistically significant, and the effect size was small, accounting for only 5% of the variability.

**Table 2:** Leptin/BMI ratio in plasma.

Factors	Level	M	n	SD	SE	Min	Max	F	*p*-value	η^2^_part._
Group	Endometriosis	0.716	40	0.259	0.041	0.275	1.376	0.59	0.4440	0.01
	Control	0.699	28	0.273	0.052	0.289	1.372			
Primary infertility	Yes	0.674	21	0.272	0.059	0.275	1.376	0.13	0.7244	<0.01
	No	0.724	47	0.260	0.038	0.289	1.372			
Interaction										
Endometriosis	Yes	0.640	18	0.278	0.066	0.275	1.376	3.56	0.0639	0.05
	No	0.778	22	0.231	0.049	0.343	1.254			
Control	Yes	0.878	3	0.103	0.059	0.767	0.969			
	No	0.678	25	0.280	0.056	0.289	1.372			

**Figure 2:**
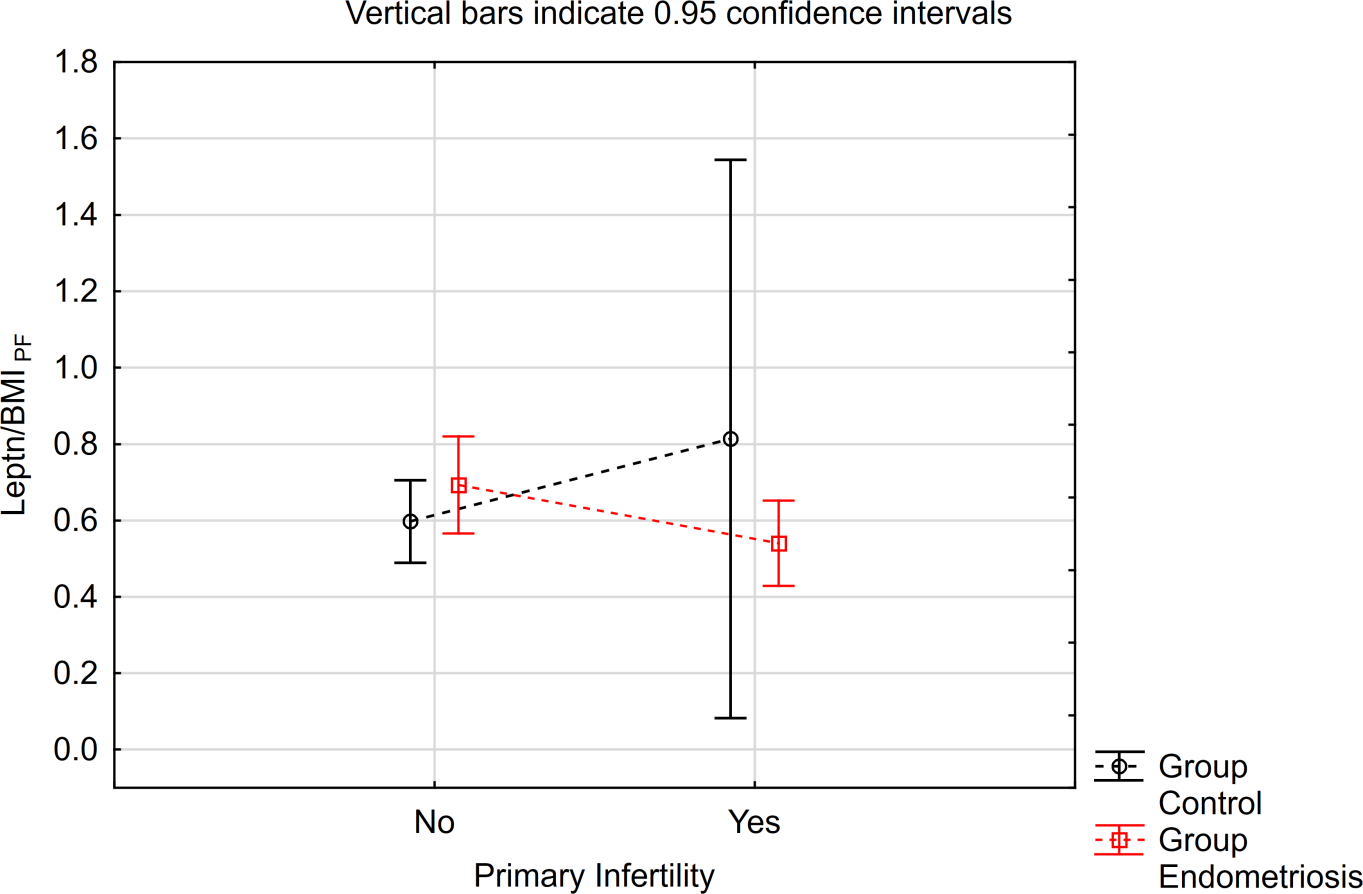
The means of leptin/BMI ratio in peritoneal fluid depending of endometriosis and primary infertility.

Similarly, concerning the leptin/BMI ratio in peritoneal fluid (PF), no significant differences were observed between women with endometriosis and the control group (**Table 3**). However, a statistically significant interaction was noted. Notably, within the primary infertility subgroup, the level of leptin/BMI in PF was significantly lower in the endometriosis group compared to the control group (**Table 3**, **Figure 2**). Conversely, in the subgroup without infertility, the level of leptin/BMI in PF was marginally higher in the study group than in the control group (**Table 3**, **Figure 2**).

**Table 3:** Leptin/BMI ratio in peritoneal fluid.

Factors	Level	M	n	SD	SE	Min	Max	F	*p-value*	η^2^_part._
Group	Endometriosis	0.621	38	0.258	0.042	0.117	1.185	0.13	0.7173	<0.01
	Control	0.621	27	0.264	0.051	0.082	1.150			
Primary infertility	Yes	0.580	21	0.247	0.054	0.117	1.150	1.02	0.3169	0.02
	No	0.641	44	0.264	0.040	0.082	1.185			
Interaction										
Endometriosis	Yes#	0.541	18	0.225	0.053	0.117	0.921	4.36	0.0409	0.07
	No	0.693	20	0.271	0.061	0.155	1.185			
Control	Yes#	0.813	3	0.173	0.100	<0.001	1.150			
	No	0.597	24	1.511	0.308	<0.001	1.041			

M, mean; n, numbers; SD, standard deviation; SE, standard error; η^2^_part._- eta_squared partial.

Means marked with # differed p<0.05 (Duncan’s test).

### Leptin/BMI ratio in endometriosis – associated infertility

Among women with endometriosis, 55% (n=22) were diagnosed with infertility (primary or secondary). In the control group, this percentage was 23% (n=7). A statistically significant relationship was observed between infertility and the health condition status, with a p-value of 0.0136. Additionally, a moderate relationship with a Yule’s coefficient (φ) of 0.31 was found (**Table 4**).

**Table 4:** Frequency distributions and percentages of fertility assessments in respective groups.

Group	Infertility		∑	*p-value*	ϕ
	Yes	No			
**Endometriosis, n(%)**	22 (55.0%)	18 (45.0%)	40 (57.1%)	**0.0136**	0,32
**Control, n(%)**	7 (23.3%)	23 (76.7%)	30 (42.9%)		
**Total, n (%)**	29 (41.4%)	41 (58.6%)	70 (100%)		

Fisher’s exact 2-tailed test was used for estimate p-value.

Bold values was to emphasize the statistical significance.

**Figure 3:**
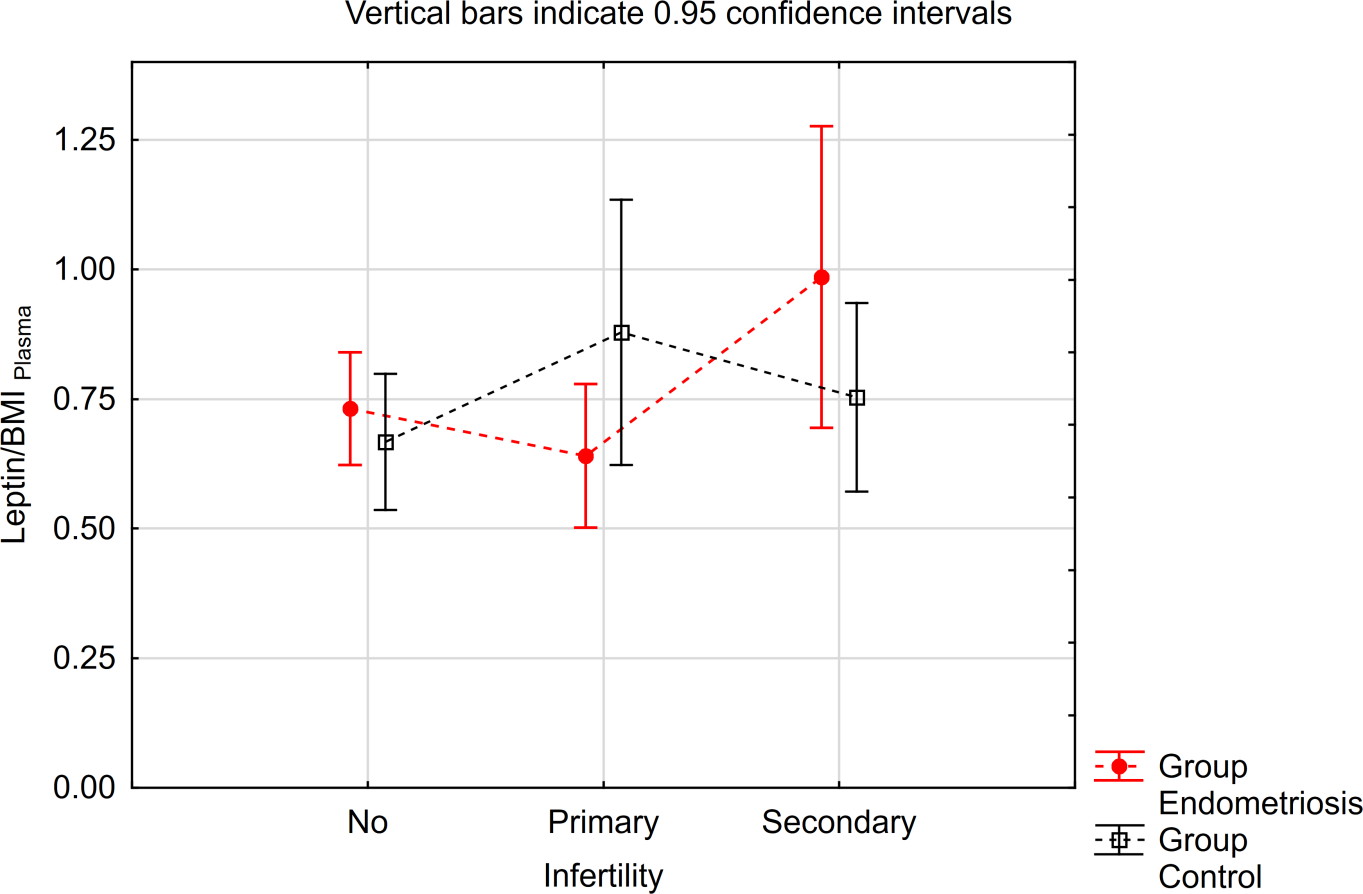
The means of leptin/BMI ratio in plasma depending of endometriosis and infertility.

**Figure 4:**
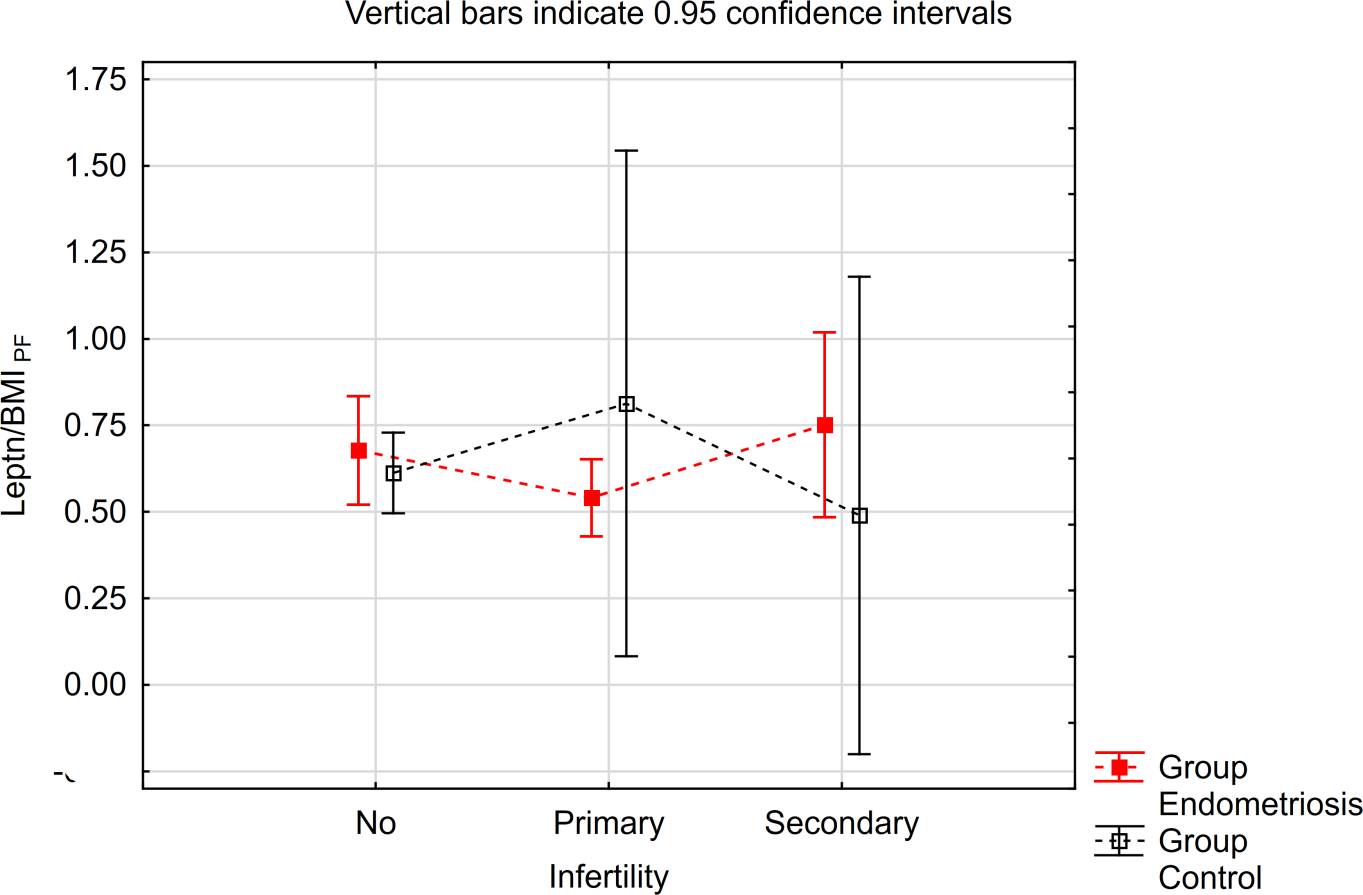
The means of leptin/BMI ratio in PE depending of endometriosis and infertility.

The contrast analysis revealed statistically significant differences in trends (quadratic polynomial) between the endometriosis group and the control group, both in the distribution of mean leptin/BMI ratio in plasma and in PF (**Table 5**, **Figures 3**, **4**). In endometriosis cases without infertility and with secondary infertility, the means were higher compared to endometriosis cases with primary infertility. Conversely, in the control group, the highest level of Leptin/BMI ratio was observed in the group with primary infertility, followed by the other subgroups. These findings indicate varying patterns of average leptin levels depending on fertility status and the type of infertility, which could contribute to a better understanding within the broader context of existing research on the subject. Nevertheless, it is crucial to note that these results should be interpreted with caution due to the relatively small size of the groups.

**Table 5:** Leptin/BMI ratio - contrast analysis (E vs C) x (trendy quadratic polynomial).

	Group	Infertility			F	*p-value*
		No M ± SE; n	Primary M ± SE; n	Secondary M ± SE; n		
**plasma**	Endometriosis	0.731 ± 0.051; 18	0.64 ± 0.066; 18	0.985 ± 0.091; 4	4.02	**0.0492**
	Control	0.667 ± 0.063; 22	0.878 ± 0.059; 3	0.753 ± 0.042; 3		
**peritoneal fluid**	Endometriosis	0.678 ± 0.073; 16	0.541 ± 0.053; 18	0.751 ± 0.084; 4	5.15	**0.0270**
	Control	0.613 ± 0.056; 21	0.813 ± 0.170; 3	0.490 ± 0.160; 3		

Bold values was to emphasize the statistical significance.

### Leptin correlation

**Figure 5:**
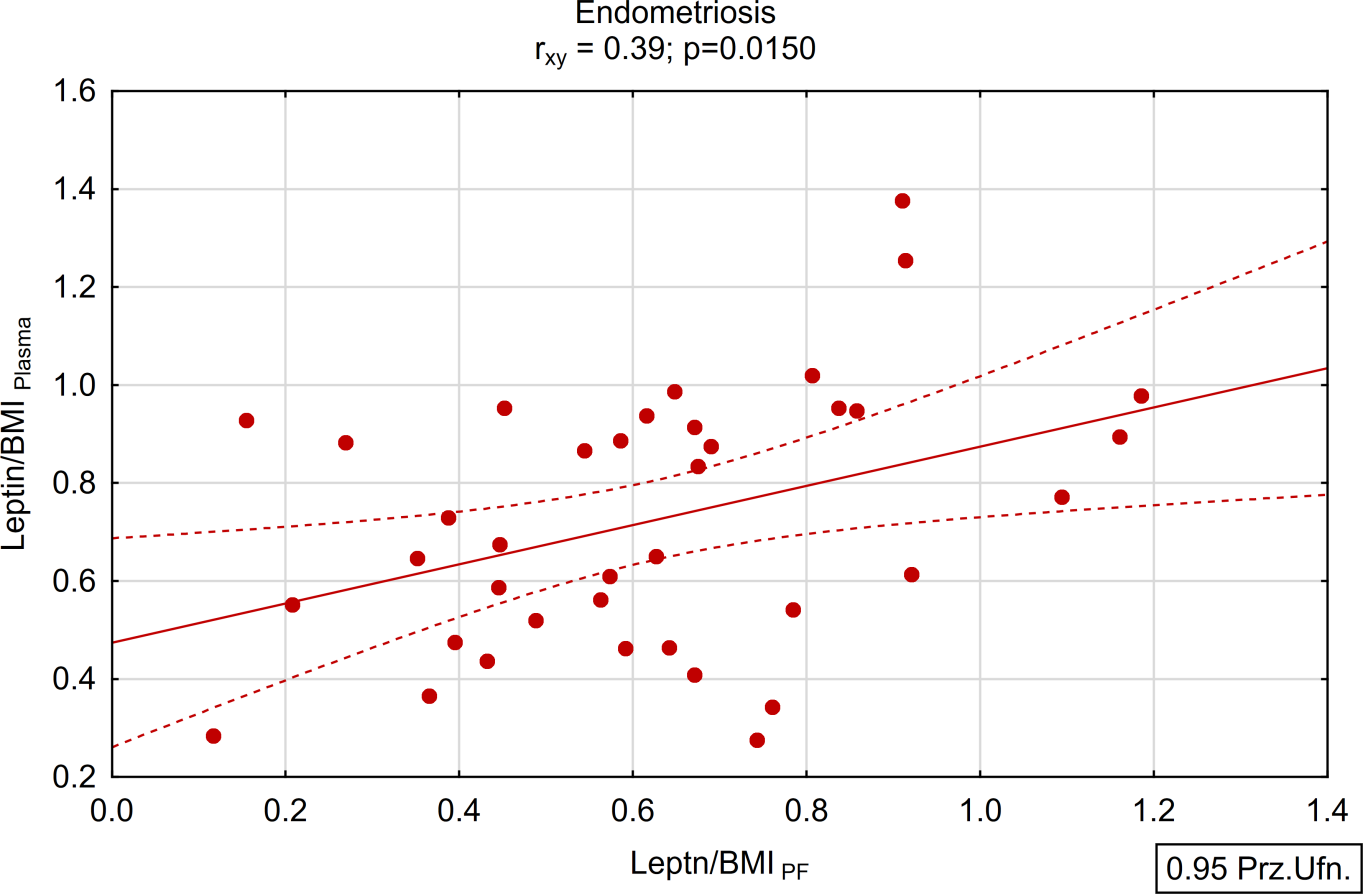
Correlations between plasma and peritoneal fluid Leptin/BMI ratio in endometriosis group (N=38).

**Figure 6:**
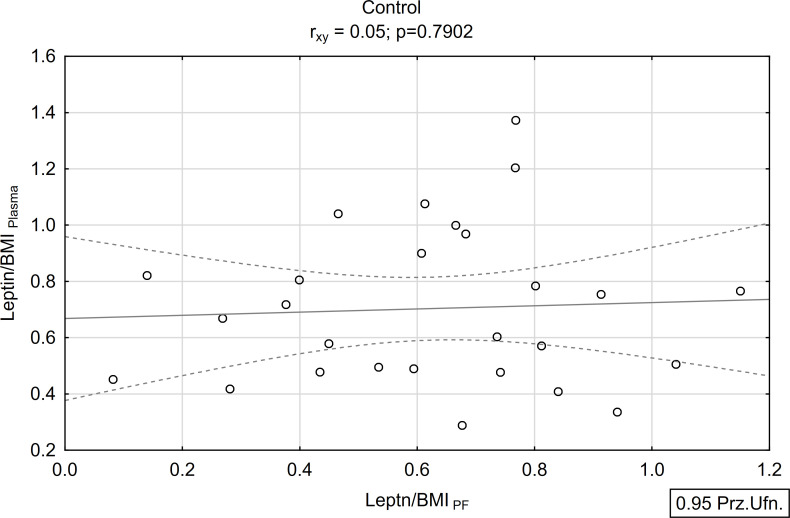
Correlations between plasma and peritoneal fluid Leptin/BMI ratio in control group (N=27).

A positive and statistically significant correlation of moderate strength was found between the concentration of leptin in peritoneal fluid and plasma within the study group (r_xy_=0.39; p<0.05; **Figures 5**, **6**). Conversely, no such association was observed in the control group (r_xy_=0.05; p>0.05; **Figures 5**, **6**).

### Leptin/BMI ratio in reference to endometriosis stage, presence of endometrioma, menstrual cycle phase

There were no significant differences observed in the leptin/BMI ratio in plasma, peritoneal fluid leptin concentration, based on the endometriosis stage, presence of endometrioma, or menstrual cycle phase (**Table 6**).

**Table 6:** Leptin/BMI ratio according to the stage of endometriosis, endometrioma and cycle phase.

Factors		M	SD	n	Min	Max	F	*p*-value	η^2^_part_.
rAFS stage of disease									
Plasma (N=39)	1	0.64	0.33	11	0.28	1.38	0,99	0,4097	0,08
	2	0.73	0.21	11	0.44	1.02			
	3	0.69	0.19	11	0.41	0.98			
	4	0.85	0.30	7	0.28	1.25			
Peritoneal fluid (N=37)	1	0.56	0.22	11	0.21	0.91	0,62	0,6047	0,05
	2	0.71	0.25	11	0.39	1.16			
	3	0.59	0.32	9	0.15	1.19			
	4	0.61	0.27	7	0.12	0.91			
Endometrioma									
**Plasma (N=68)**	No	0.70	0.27	45	0.28	1.38	0.32	0.7507	<0.01
	Yes	0.72	0.25	23	0.28	1.25			
Peritoneal fluid (N=65)	No	0.60	0.26	44	0.08	1.16	0.81	0.4232	0.01
	Yes	0.66	0.26	21	0.12	1.19			
Cycle phase									
**Plasma (N=68)**	proliferative	0.72	0.25	44	0.28	1.37	-0.28	0.7785	<0.01
	secretive	0.70	0.29	24	0.28	1.38			
Peritoneal fluid (N=65)	proliferative	0.61	0.27	42	0.08	1.16	0.35	0.7306	<0.01
	secretive	0.64	0.24	23	0.27	1.19			

**Figure 7:**
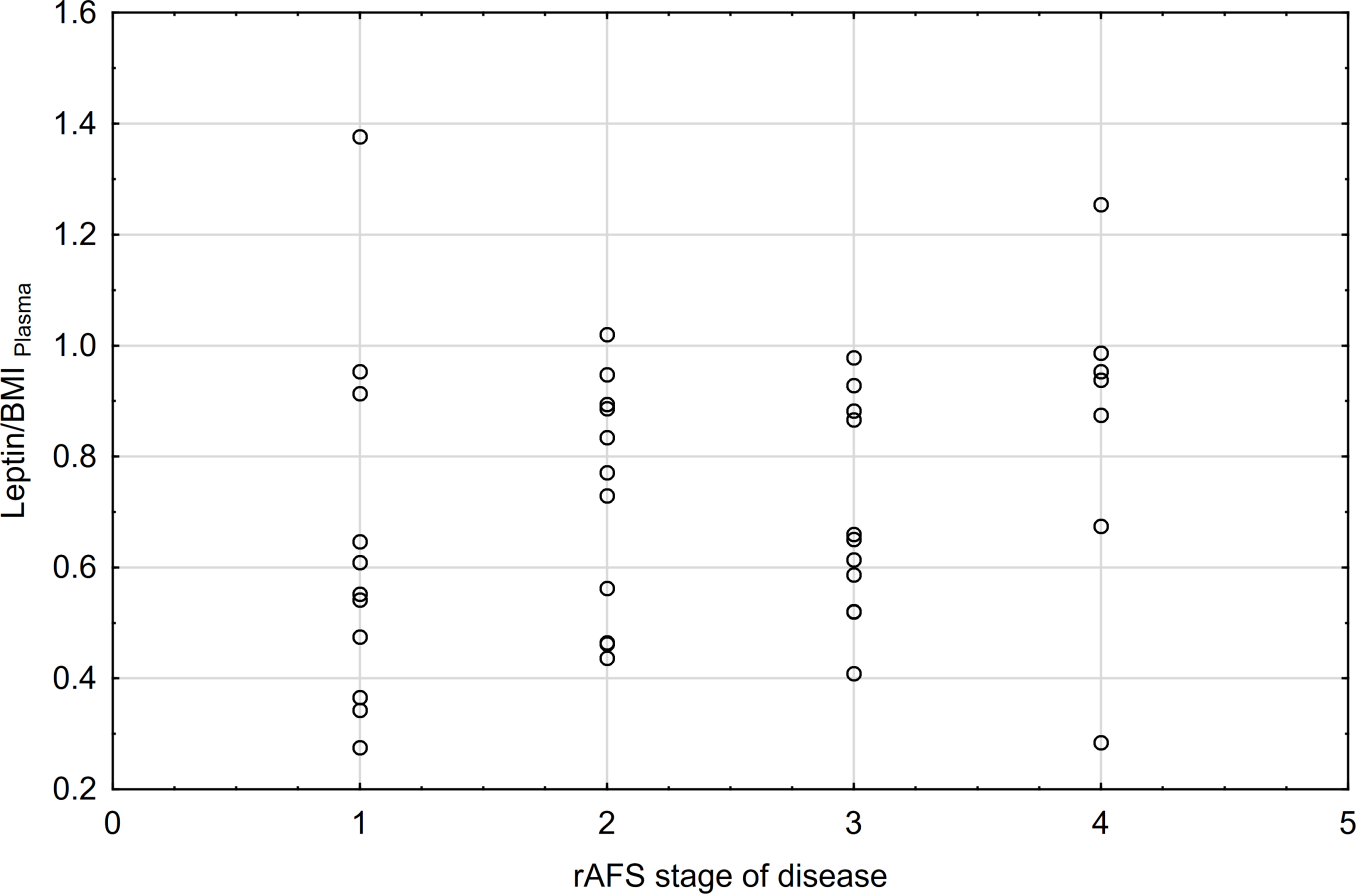
Correlations between rAFS stage of disease & Leptin/BMI ratio in plasma (rs=0.28; p=0.0871; N=39).

**Figure 8:**
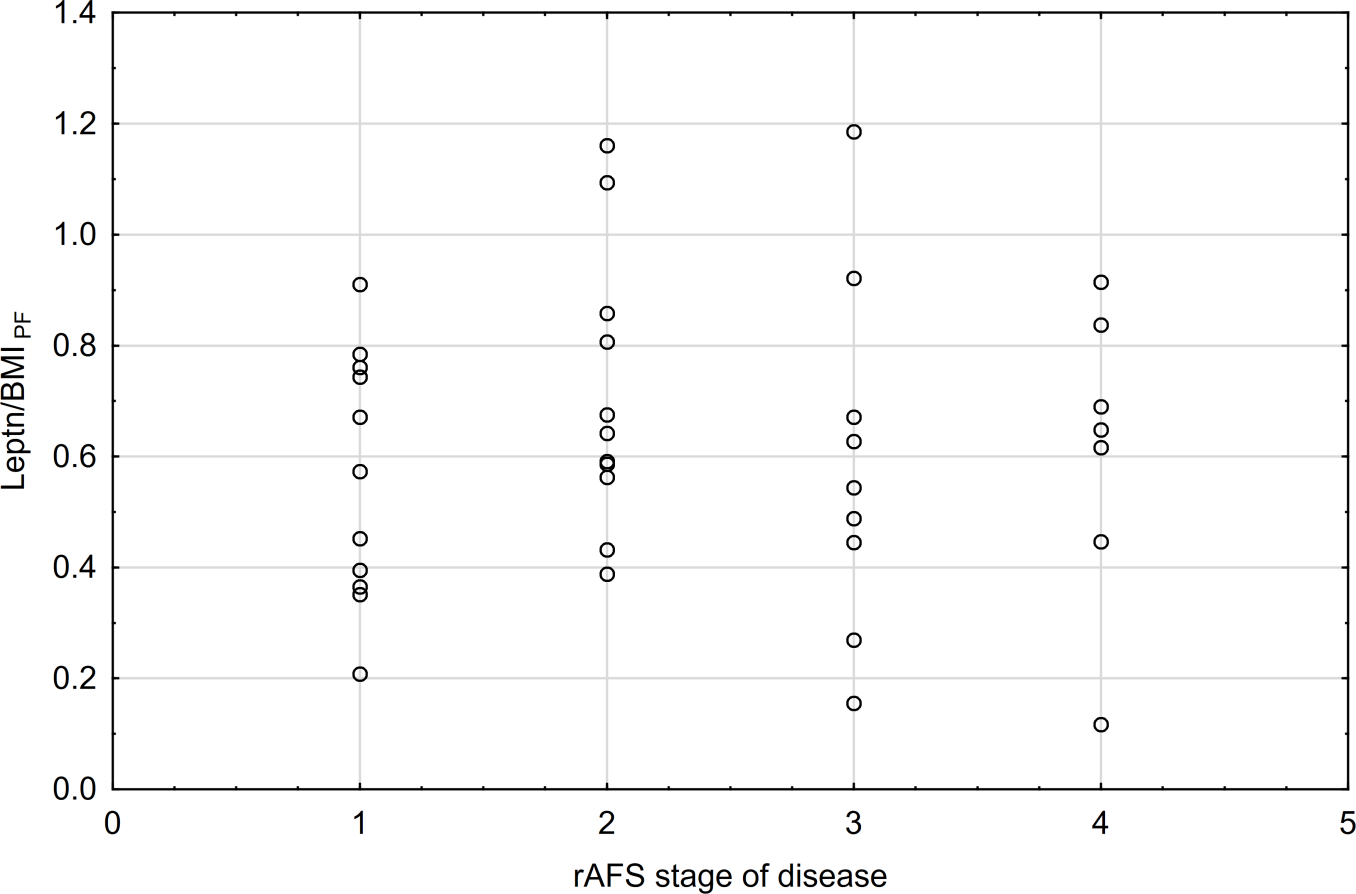
rAFS stage of disease & Leptin/BMI ratio in PF (rs=0.05; p=0.7570; N=37).

The correlations between rAFS stage of disease & leptin/BMI ratio in plasma and rAFS stage of disease & leptin/BMI ratio in peritoneal fluid (PF) were not found (**Figures 7**, **8**).

## Discussion

In the present study, we found that plasma and peritoneal leptin concentrations did not differ between the studied groups. Furthermore, after adjusting for BMI in both the endometriosis and control groups, no significant difference was observed in leptin level between the groups, whether analyzing peritoneal or serum leptin concentrations.

These findings align with Wertel et al.’s study, supporting our thesis (42). However, Rathore et al. reported contrasting results, noting significantly higher leptin levels in peritoneal fluid among women with endometriosis compared to the control group, while serum leptin levels did not differ between the groups (43).

Interestingly, in a previous clinical study positive correlation between PF and serum leptin concentrations was found among fertile patients with endometriosis (42). Moreover, another research study found no significant difference in peritoneal leptin levels between the endometriosis-associated infertility group and those with fallopian-associated infertility (44). These results are consistent with our study, which revealed that a subgroup of women with endometriosis related infertility had a significantly lower concentration of leptin adjusted for BMI in PE compared to women with endometriosis, but without primary infertility. However, it’s important to note that the subgroups of patients with infertility in our study were small, so result may not lead to reliable premise. In order to overcome this limitation, further cooperation and research by a multi-center working group on endometriosis is planned. Hence, at present, we can only hypothesize that leptin might contribute to pelvic inflammation in endometriosis, potentially altering the local microenvironment of the peritoneal cavity, yet its direct impact on endometriosis-associated infertility is uncertain.

As previously mentioned, studies evaluating leptin levels in serum and peritoneal fluid among patients with endometrioma have shown conflicting results, reporting either elevated levels or no significant changes. In our study, we found no significant difference in both serum and PF leptin concentrations concerning the presence of endometrioma. Consistent, with our findings, Zendron et al. noted comparable leptin concentrations between patients with endometrioma and those in the control group (45).

However, recent research has revealed higher peritoneal fluid leptin concentrations among women displaying peritoneal lesions compared to those with endometrioma as the only finding (46). These differences observed among diverse phenotypes of the disease such as peritoneal versus ovarian endometriosis, suggest a potential role of leptin in the development of peritoneal endometriosis. This implies that different biochemical phenomena might be involved in the pathogenesis of the ovarian form of the disease.

Fluctuations in leptin concentration during the menstrual cycle remain a subject of controversy. Many studies have noted higher leptin levels in the midluteal phase compared to the follicular phase (47, 48). It is suggested that estradiol and progesterone may play an essential role in regulating leptin release during cycle. Ajala et al. demonstrated a significant increase in serum leptin levels on both day 14 (ovulatory phase) and day 21 (luteal phase) of the menstrual cycle (47). In comparison, another study noted higher leptin concentrations during midluteal phase (49), with observed positive correlations between leptin values and estradiol or progesterone levels (50). Conversely, our study findings indicated no significant changes in leptin/BMI ratio throughout the menstrual cycle. These result align with Stock et al.’s study, which similarly found no correlation between leptin values and estradiol or progesterone levels (51). Furthermore, Capobianco’s research revealed no significant differences in leptin levels during the menstrual cycle (52). It needs to be underlined that the aforementioned studies primarily focused on either healthy women or women with infertility problems, but not specifically on those with endometriosis. Considering numerous clinical reports highlighting aberrant progesterone signaling in patients with endometriosis, it is plausible that such regulation might be altered or missed in the context endometriosis (53).

Worth to note is that, a positive and statistically significant correlation of moderate strength was observed between the concentration of leptin in peritoneal fluid and plasma within the endometriosis group. These findings imply that plasma may serve as a viable alternative for assessing leptin concentration among women with endometriosis, presenting a less invasive method for evaluation.

However, we acknowledge limitation in this study. The relatively small sample size of both the study and the control groups poses a constraint. To address this, further studies based on a multicenter patient population in Poland are already planned, aiming to broaden and extend the analysis.

A strength of our study lies in the utilization of SPRI, a well-established and reliable technology for measurements. Additionally, through the assessment of both leptin concentrations in plasma and peritoneal fluid, we investigated their interrelationships and potential impact on the pathogenesis of endometriosis. Another advantage of our research was the meticulous sampling procedure, emphasizing the purity of the collected peritoneal fluid. Significantly, our study contributes to a series of publications examining the potential role of selected molecules as biomarkers for endometriosis.

In conclusion, the role of leptin as a reliable biomarker of endometriosis remains controversial. Our study aligns with the thesis, that peritoneal fluid and serum leptin level may not significantly differ between patients with endometriosis and the control group. However, our findings suggest an association between leptin concentration and endometriosis-associated infertility, albeit based on a limited number of samples. Hence, further research is imperative to elucidate the role of leptin in endometriosis.

Further studies are needed to delve into the underlying mechanisms through which leptin operates in endometriosis, determining whether observed changes in leptin concentrations contribute to or are the result from the disease’s pathogenesis. Additionally, more comprehensive data regarding leptin as a diagnostic biomarker are necessary for a clearer understanding of its potential utility in the diagnostic process of endometriosis.

## Data availability statement

The original contributions presented in the study are included in the article/supplementary material. Further inquiries can be directed to the corresponding author.

## Ethics statement

The studies involving humans were approved by Ethics Committee of the Medical University of Warsaw (KB/223/2017). The studies were conducted in accordance with the local legislation and institutional requirements. The participants provided their written informed consent to participate in this study.

## Author contributions

AZ: Visualization, Validation, Formal analysis, Conceptualization, Writing – original draft. AnS: Writing – review & editing, Methodology. AgS: Writing – review & editing, Supervision. ED: Writing – review & editing. AD: Writing – review & editing, Resources. GM: Writing – review & editing, Resources. MKi: Writing – review & editing, Resources. RS: Writing – review & editing, Resources. PPk: Writing – review & editing, Resources. BB: Writing – review & editing, Resources. AJ: Writing – review & editing, Resources. TI: Writing – review & editing, Resources. WR: Writing – review & editing, Resources. JM: Writing – review & editing, Resources. MS: Writing – review & editing, Resources. PS: Resources, Writing – review & editing. GR: Writing – review & editing, Resources. KS: Writing – review & editing, Resources. TK: Writing – review & editing, Resources. MKl: Resources, Writing – review & editing. PPz: Writing – review & editing, Resources. CW: Writing – review & editing, Resources. ML: Writing – review & editing, Supervision, Methodology. DW: Writing – review & editing, Resources. MW: Writing – review & editing, Funding acquisition. KC: Writing – review & editing, Resources. EG: Writing – review & editing, Methodology. PL: Writing – review & editing, Supervision, Funding acquisition, Formal analysis, Conceptualization.
